# Mathematical Methods for Images and Surfaces 2011

**DOI:** 10.1155/2012/419647

**Published:** 2012-03-21

**Authors:** Weihong Guo, Lalita Udpa, Yang Wang, Guowei Wei, Shan Zhao

**Affiliations:** ^1^Department of Mathematics, Case Western Reserve University, Cleveland, OH 44106, USA; ^2^Department of Electrical and Computer Engineering, Michigan State University, East Lansing, MI 48824, USA; ^3^Department of Mathematics, Michigan State University, East Lansing, MI 48824, USA; ^4^Department of Mathematics, University of Alabama, Tuscaloosa, AL 35487, USA

The study of biomedical imaging and biological surfaces is a rapidly growing interdisciplinary field that has attracted considerable interest from mathematical, engineering, and medicine communities. Many research problems in this field are application oriented and thus the results have practical values but are challenging due to physical and biological constraints and the large scale nature of massive biomedical and biomolecular data. To efficiently solve these problems, advanced mathematical models and fast and efficient computational algorithms are indispensable tools. This special issue was called to address mathematical difficulties and challenges in image and surface analysis. We would like to share with the readers the recent advances in topics such as topological-gradients-based edge/contour detection, partial differential-equation-transform-based feature separation, blind multiple-source reconstruction in bioluminescence tomography, partial-differential-equation-based cerebral cortex reconstruction, nonlinear elasto-mammography for characterization of breast tissue properties, and protein surface characterization.

We would like to thank the authors for their excellent contributions and patience that make this special issue possible. The time, effort, and valuable work of all anonymous reviewers on these papers are also very greatly acknowledged. This special issue constitutes ten papers.

The paper entitled “*Contour detection and completion for inpainting and segmentation based on topological gradient and fast marching algorithms*” by D. Auroux et al. introduces a contour functional that is based on topological gradient and couples it with a fast marching algorithm to determine the minimal path for the purpose of generating connected contours. This offers a hybrid scheme for edge detection and contour completion. Two specific applications are considered for image processing. For image segmentation, the topological gradient is shown to be more efficient than the standard gradient approaches. For image inpainting, the hybrid scheme particularly improves the quality of the inpainted images.

The paper entitled “*Protein surface characterization using an invariant descriptor*” by Z. Abu Deeb et al. develops a new invariant descriptor for the characterization of protein surfaces. It is suitable for various analysis tasks, such as protein functional classification and search and retrieval of protein surfaces over a large database. Its novelty is the combination of the power of residue-distance cooccurrence-based local and global surface descriptors. The proposed method not only reduces the computational complexity of matching 3D structures, but also facilitates direct comparison between protein structures of different sizes. The comparison with other methods on three protein families indicates that this method is effective.

The paper entitled “*Extending local canonical correlation analysis to handle general linear contrasts for fMRI data*” by M. Jin et al. designs a novel test statistics to enable canonical correlation analysis (CCA) and to handle general linear contrasts in more complicated fMRI paradigms. This approach avoids the reparameterization of the design matrix and the reestimation of the CCA solutions for each particular contrast of interest. This test statistics is more powerful than the traditional *t*-test in general linear models on the inference of evoked brain regional activations from noisy fMRI data, especially for weakly evoked and localized brain activations. The method improves detection power with acceptable computation time and has potential to meet the needs in recent fMRI where data is enormous, signal is weak, and the spatial correlation is strong.

The paper entitled “*A novel FEM-based numerical solver for interactive catheter simulation in virtual catheterization*” by S. Li et al. concerns with interactive simulation of the deformable catheters and guide-wires in virtual vascular interventional surgeries. The motion of catheters or guide-wires and their interactions with patients' vascular system are mathematically formulated in terms of a total potential energy, consisting of bending elastic energy, vessel wall deformation energy, and the work by the external forces. The minimization of the potential energy functional is numerically realized via a finite element simulation. Experimental studies indicate that the proposed method can realistically model and simulate deformable catheters and guide-wires in an interactive manner.

The paper entitled “*Cortical surface reconstruction from high-resolution MR brain images*” by S. Osechinskiy et al. presents a new PDE-based approach that readily scales with imaging resolution for reconstructing the cerebral cortex from MR images. The scalability virtue of the approach makes it promising in brain imaging research where high-resolution MRI becomes more popular. This scalability is achieved by using an implicit deformable surface model in a fast marching framework guided by a novel, computationally efficient model using potential field mapping. The method requires much lower computational resources and allows much faster computations than conventional methods.

The paper entitled “*Serial FEM/XFEM-based update of preoperative brain images using intraoperative MRI*” by L. Vigneron et al. aims to overcome the limitation of current neuronavigation systems that cannot adapt to changing intraoperative conditions over time. The authors develop a complete 3D framework for serial preoperative images updated in the presence of brain shift followed by successive resections. The key ingredient of the system is a nonrigid registration technique using a biomechanical model driven by the deformations of key surfaces tracked in successive intraoperative images. Numerical results demonstrate that the present approach significantly improves the alignment of nonrigidly registered images.

The paper entitled “*Selective extraction of entangled textures via adaptive PDE transform*” by Y. Wang et al. presents a new adaptive algorithm for selective extraction of entangled textures. Texture characterization and analysis are complicated for images with spatial entanglement, orientation mixing, and high-frequency overlapping. Based on a recently developed PDE transform method for functional mode decomposition, the statistical variance of the local variation is adaptively incorporated in the PDE transform framework for separating textures of very similar features. Successful texture separation is attained for several benchmark images.

The paper entitled “*Nonlinear elasto-mammography for characterization of breast tissue properties*” by Z. G. Wang et al. extends their previous studies by incorporating the projection of displace information obtained from the conventional X-ray mammography into a nonlinear elastography framework. In particular, projection-type displacement measurements are considered before and after breast compression, and a revised adjoint gradient method is derived for calculating the gradient of the objective function in the nonlinear elasto-mammography framework. Simulations based on a three-dimensional breast phantom involving normal and cancerous tissues are conducted to validate the feasibility and robustness of the proposed approach.

The paper entitled “*Fracture detection in traumatic pelvic CT images*” by J. Wu et al. presents an automated hierarchical algorithm for bone fracture detection in pelvic CT scans. It uses adaptive windowing, boundary tracing, and wavelet transform, while incorporating anatomical information. Fracture detection is performed based on the results of prior pelvic bone segmentation via their registered active shape model (RASM). The results are promising and show that the method is capable of detecting fractures accurately. Once verified with more data, the proposed method has the potential to be an important component of a larger modular system to extract features from CT images for a computer-assisted decision making system.

The paper entitled “*A finite element mesh aggregating approach to multiple-source reconstruction in bioluminescence tomography*” by J. Yu et al. develops a finite element mesh aggregating algorithm for blind multiple-source reconstruction in bioluminescence tomography. Without knowing the number of the sources in advance, an iterative procedure is utilized to detect multiple sources by exploiting the spatial structure of the nodes in finite element meshes and the characteristics of the energy decay. The detecting algorithm is formulated in a flexible reconstruction framework, where a variety of regularizers and inversion algorithms can be chosen by the user. Simulations using a tissue-like phantom demonstrate an improved performance of the new algorithm in terms of the automatic estimation of both locations and densities of multiple sources that differ greatly in power.

*Weihong Guo* *Weihong Guo* *Lalita Udpa* *Lalita Udpa* *Yang Wang* *Yang Wang* *Guowei Wei* *Guowei Wei* *Shan Zhao* *Shan Zhao*

